# Nonlinear Feature Modeling Using Kolmogorov-Arnold Networks in Electroencephalography for Depression Classification: Deep Learning Study

**DOI:** 10.2196/101076

**Published:** 2026-10-19

**Authors:** Bin Yang, Yongfeng Fan, Xinhong Wei, Chunlei Shi

**Affiliations:** 1 Henan University of Urban Construction, No. 207, Longxiang Avenue, Xincheng District, Pingdingshan, Henan, 467000, China, 86 13837588771

**Keywords:** depression, electroencephalography, diagnosis, computer-assisted, deep learning, neural networks, computer, signal processing, computer-assisted, pattern recognition, automated

## Abstract

**Background:**

Depression is a common mental disorder that seriously affects mental health and quality of life. Traditional depression diagnosis mainly relies on clinical interviews and subjective assessments, which may lead to variability in diagnostic outcomes. Electroencephalogram (EEG)-based computer-aided diagnosis has emerged as a promising, objective approach for depression recognition. However, effectively modeling the complex nonlinear characteristics of EEG signals remains a major challenge for existing deep learning methods.

**Objective:**

This study aimed to investigate the applicability of Kolmogorov-Arnold Networks (KANs) for EEG-based depression recognition and to evaluate the effectiveness of KAN-based nonlinear modeling frameworks on frequency-domain EEG features.

**Methods:**

KAN was introduced into EEG-based depression recognition to enhance nonlinear feature representation of brain electrical signals. Two representative KAN-based convolutional architectures, ConvKAN and its efficiency-optimized variant ConvKAN Efficient, were systematically investigated for EEG classification tasks. These architectures incorporated KANLinear layers together with dynamic grid updating, enhanced weight initialization, and regularization strategies to improve nonlinear modeling capability while maintaining computational efficiency. Extensive experiments were conducted on the EDRA dataset, and the proposed framework was compared with conventional convolutional neural network–based methods and traditional machine learning approaches. Model performance was evaluated using accuracy, precision, recall, and *F*_1_-score metrics.

**Results:**

Experimental results demonstrated that the KAN-based frameworks achieved superior EEG classification performance compared with conventional convolutional neural network–based methods and traditional machine learning approaches. Among the evaluated models, ConvKAN Efficient achieved the best overall performance, with accuracy, precision, recall, and *F*_1_-score all exceeding 94% on the EDRA dataset. The results indicate that KAN-based architectures can effectively capture the complex nonlinear characteristics of EEG signals while preserving high computational efficiency.

**Conclusions:**

This study systematically demonstrates the applicability of KAN-based nonlinear modeling frameworks to EEG-based depression recognition. The proposed framework provides a scalable and effective approach for EEG signal analysis and offers valuable technical support for both theoretical research and clinical applications in computer-aided depression recognition.

## Introduction

### Background and Motivation

Depression is a common mental disorder that affects hundreds of millions of people worldwide and represents one of the leading causes of disability and suicide [1,2]. According to statistics from the World Health Organization, depression has become a major contributor to the global burden of disease, exerting profound negative impacts on patients’ quality of life and social functioning [3]. At present, the diagnosis of depression primarily relies on clinical interviews and self-reported symptoms, which are inherently subjective and uncertain, making early diagnosis particularly challenging. To address these limitations, an increasing number of studies have explored the application of electroencephalogram (EEG) in depression recognition. EEG enables real-time recording of brain electrical activity and reveals abnormal neural patterns associated with depression, thereby providing valuable objective evidence for clinical assessment [4]. However, due to the complexity of EEG signals and their susceptibility to noise, accurately extracting discriminative features and achieving efficient classification remain significant challenges.

In recent years, deep learning has emerged as a dominant approach for EEG signal analysis and has been widely applied to depression classification tasks. Existing studies indicate that models integrating graph convolutional networks with attention mechanisms can effectively capture interchannel correlations in the EEG signals as well as cross-participant distribution discrepancies, thereby improving model generalization performance [5]. Meanwhile, region-of-interest–based analyses have shown that depression exhibits significant characteristics in specific brain regions and frequency bands, and nonlinear features such as sample entropy and approximate entropy can effectively distinguish patients with depression from healthy controls [6]. Building upon these findings, deep models that fuse temporal, spectral, and spatial information have further enhanced EEG feature representations, achieving more stable classification performance in depression recognition tasks [7].

Subsequent studies have introduced multiscale and dynamic modeling strategies. By combining dynamic graph convolutional networks with spiking neural networks (SNNs), these approaches strengthen the modeling capability of multichannel EEG topological structures and their nonlinear dynamics for depression recognition [8]. At the same time, densely connected networks based on self-attention mechanisms have enabled automatic depression detection through multisource feature fusion, validating the effectiveness of attention mechanisms in this task [9]. In addition, time-frequency proxy graph learning methods model cooperative relationships among EEG signals at cross-regional and whole-brain levels, providing novel perspectives for depression classification [10]. Deep learning frameworks based on resting-state EEG signals have also been used to distinguish multiple affective disorders, further expanding the application scope of EEG-based analysis [11].

Against this background, this study proposes an EEG-based depression recognition method grounded in the Kolmogorov-Arnold Network (KAN) [12]. KAN is a neural network model inspired by nonlinear dynamical systems theory and is capable of capturing complex data structures and latent patterns, demonstrating superior performance in complex signal processing tasks. Although KAN has shown promising results in domains such as image recognition and speech processing, its application to EEG signal analysis remains largely unexplored, particularly in the context of depression prediction. Therefore, one of the key contributions of this work is the introduction of KAN into EEG-based depression recognition, aiming to more effectively mine depression-related features from EEG signals and improve classification accuracy and model robustness.

### Related Work

#### Machine Learning–Based Depression Recognition

In recent years, machine learning techniques have achieved remarkable progress in depression recognition, with research primarily focusing on text analysis, behavioral data, and multimodal prediction. For social media text analysis, Khan and Alqahtani [13] proposed several hybrid machine learning models to perform sentiment analysis on X (formerly known as Twitter; X Corp) posts for early depression screening, achieving an accuracy of up to 0.994 using TF-IDF (term frequency–inverse document frequency) features combined with logistic regression (LR). Khan and Gupta [14] introduced an ensemble learning–based early risk detection framework that integrates LR, decision trees (DTs), random forests, support vector machines (SVMs), multilayer perceptrons, and gradient boosting methods to effectively identify individuals at risk of depression. Ayodele et al [15] used multimodal data, including demographic, lifestyle, and clinical information, to predict individualized depression severity using models such as random forest and XGBoost (Extreme Gradient Boosting), providing a basis for personalized risk factor analysis. In the field of neuroimaging, Zhou et al [16] used multimodal brain imaging features combined with a random forest model to predict anxious depression, achieving an area under the curve (AUC) of 0.802 in distinguishing anxious from nonanxious depression. Furthermore, Upadhyay et al [17] proposed a stacked SVM approach for persistent depressive disorder, modeling behavioral data from university students and achieving an accuracy of 89.4% for early assessment.

In physiological signal analysis, EEG has become an important modality for machine learning–assisted depression recognition. Hassan et al [18] systematically compared various feature selection techniques and classifiers, demonstrating that the combination of SVM-RFE (support vector machine–recursive feature elimination) and SVM achieved the best performance in EEG-based depression recognition, with an accuracy of 93.54%. Bashir et al [19] developed a multimodel framework based on EEG features, in which k-nearest neighbors and long short-term memory (LSTM) achieved accuracies of 87.5% and 83.3%, respectively, supporting multistage early intervention for major depressive disorder (MDD). Earl et al [20] used EEG functional connectivity under resting-state and emotional stimulation conditions for MDD diagnosis, with a random forest model achieving a cross-validation accuracy of 94.9%. Suzuki et al [21] used consumer-grade EEG devices combined with a LightGBM model to detect depression, attaining an *F*_1_-score of 91.59% and validating the clinical potential of low-cost EEG systems. Balakrishna et al [22] integrated EEG data with clinical and demographic information and applied CatBoost and XGBoost for hybrid modeling, achieving a prediction accuracy of 93.1% for MDD. Dhekane and Khandare [23] optimized EEG channel selection and used a multilayer perceptron model, achieving an accuracy of 98.7% and an *F*_1_-score of 0.983, providing a highly reliable method for depression recognition.

Machine learning–based approaches for depression recognition span multiple data sources, including text, behavioral data, neuroimaging, and EEG signals. Hybrid models, ensemble learning strategies, and feature selection optimization play crucial roles in improving early detection and diagnostic accuracy, thereby laying a solid theoretical and practical foundation for personalized intervention and clinical decision-making.

#### Deep Learning-Based Depression Recognition

With the widespread adoption of deep learning in medical signal analysis, EEG has emerged as a critical objective indicator for depression recognition. In recent years, numerous studies have leveraged deep neural networks to extract spatiotemporal and spectral features from EEG signals, thereby improving the accuracy and robustness of depression recognition. Wan et al [24] proposed the M-MDD framework, which uses multitask learning to achieve noise-robust feature learning and time-frequency-spatial feature extraction, achieving state-of-the-art performance on 2 public MDD datasets. Abir et al [25] compared traditional machine learning and deep learning approaches for EEG-based depression prediction, demonstrating that convolutional neural networks (CNNs) outperform conventional methods in complex feature extraction and classification, with accuracy exceeding 92%, albeit at the cost of higher computational complexity, suggesting the potential of hybrid approaches to balance performance and efficiency. Ahire [26] evaluated 1D-CNN, SVM, and LR models on task-related, eyes-closed, and eyes-open EEG signals, with the 1D-CNN achieving an accuracy of 90.21% on task-related signals, confirming the robustness of deep learning across different experimental conditions.

Lightweight architectures and attention mechanisms have also shown significant promise in EEG-based depression recognition. Hou et al [27] proposed the lightweight convolutional transformer neural network model, which combines CNNs and transformers to jointly learn local and global features while reducing computational complexity through channel modulators and sparse attention, achieving high cross-dataset classification performance. Kumar et al [28] introduced the EEG-ViLSTM model, integrating visual features with LSTM networks to enhance cross-participant generalization, achieving an accuracy of 93.52%. Gupta et al [29] compared UNet with a bat-optimization-based UNet, demonstrating superior noise suppression and classification accuracy of bat-optimization-based UNet over conventional UNet architectures. Rahmati et al [30] combined microstate and entropy features with CNNs and deep neural networks for noninvasive MDD diagnosis, achieving an accuracy of 99.6% under 5-fold cross-validation, highlighting the importance of nonlinear features and multimodal representations in EEG-based depression recognition.

The incorporation of spatial structural information and graph neural networks has further strengthened diagnostic performance. Ren and Song [31] proposed the P-I-GAT model, which integrates individual- and population-level brain networks using graph attention networks to achieve depression severity classification, obtaining an accuracy of 88.57% and a specificity of 91.97% on the MODMA dataset. Xi et al [32] developed a multiscale convolutional attention network that fuses spatiotemporal-spectral features and optimizes channel selection, achieving an accuracy of 97.24% on the MODMA dataset. Gan et al [33] proposed the TSF-MDD model, which integrates 3D-CNN and CapsNet to comprehensively extract temporal, spatial, and frequency-domain features while preventing data leakage through participant-independent segmentation, achieving an accuracy of 92.1%.

SNNs and lightweight models have also demonstrated strong performance in wearable EEG applications. The leaky integrate-and-fire spiking neural network by Sahu [34] uses spike sequences and a 3-layer neural architecture for depression recognition, achieving accuracy close to 98%. The LSNNet by Zhao et al [35] operates on STM32 and GAP8 microcontrollers while maintaining a classification accuracy of 89.2%, indicating strong potential for edge-device deployment. S et al [36] combined the Superlet transform with ResNet-18 to convert EEG signals into images for deep feature extraction, achieving an accuracy of 95.65%. Shen et al [37] proposed UA-DAAN, an uncertainty-aware dynamic adversarial adaptation network that improves robustness and reliability by optimizing feature transfer between source and target domains. Deng et al [38] introduced AttenStateNet, which integrates state-space models with attention mechanisms to extract long-term EEG dependencies and adaptively adjust channel importance, achieving accuracies of 99.87% and 99.67% on the MODMA and EDRA datasets, respectively.

Deep learning–based EEG depression recognition encompasses diverse technical paradigms, including CNNs, transformers, LSTMs, CapsNet, SNNs, graph neural networks, and attention mechanisms. Through multitask learning, feature fusion, channel selection, and lightweight design, these approaches have achieved significant improvements in diagnostic accuracy, robustness, and cross-participant generalization, providing a solid technical foundation for EEG-assisted clinical depression recognition and laying the theoretical groundwork for subsequent KAN-based research.

Recent studies have shown that robust nonlinear modeling, regularization constraints, and feature fusion strategies are effective for complex and limited-sample classification tasks. Wang et al [39] proposed a distribution entropy regularized multimodal subspace support vector data description method, which improves anomaly detection by combining entropy-based sample weighting, multimodal graph regularization, and sparse projection constraints. Wang et al [40] also developed a knowledge correction and ε-insensitive 0-order Takagi-Sugeno-Kang fuzzy system for rice leaf disease diagnosis, enhancing interpretability, robustness, and generalization under insufficient samples and noise. Furthermore, Wang proposed a knowledge calibration fusion and label space graph regularization-based multicenter Takagi-Sugeno-Kang fuzzy system, demonstrating the effectiveness of knowledge transfer and graph regularization for heterogeneous data integration [41]. In medical image analysis, MMFNet integrates multimodal information through multiattention fusion for multitask skin disease classification [42]. In hyperspectral image recognition, Cai et al [43] introduced a multisource patch feature fusion framework with a neighborhood flash attention transformer to enhance spectral-spatial representation learning. These studies provide useful methodological references for nonlinear feature modeling, regularized learning, and adaptive feature fusion. Different from these approaches, this study focuses on EEG-based depression recognition and investigates KAN-based convolutional architectures for modeling power spectral density (PSD)–based frequency-domain EEG features.

### Contributions of This Study

The main contributions of this study are summarized as follows:

1. KAN is introduced into EEG-based depression recognition, enabling effective modeling of complex nonlinear characteristics in brain electrical signals and providing a new perspective for EEG feature representation and analysis.
2. Two representative KAN-based convolutional architectures, ConvKAN and its efficiency-optimized variant, ConvKAN Efficient, are systematically investigated for EEG classification tasks. By integrating KANLinear layers together with dynamic grid updating, enhanced weight initialization, and regularization strategies, these architectures demonstrate strong nonlinear representation capability while preserving computational efficiency.
3. Extensive experiments conducted on the EDRA dataset demonstrate that ConvKAN Efficient achieves high-precision EEG classification performance, with accuracy, precision, recall, and *F*_1_-score all exceeding 94%, outperforming conventional CNN-based methods and traditional machine learning approaches.
4. A scalable nonlinear deep-learning framework for EEG signal analysis is established, providing valuable technical support for both theoretical studies and clinical applications in computer-aided depression recognition.

Unlike previous studies that mainly focused on CNNs, transformers, or graph neural networks, this study systematically investigates the applicability of KAN-based nonlinear modeling frameworks to EEG-based depression recognition and evaluates their effectiveness on frequency-domain EEG features.

## Methods

### Dataset and EEG Signal Preprocessing

#### Depression EEG Dataset

The EDRA dataset [44] used in this study consists of EEG recordings from 50 university students, among whom 26 participants are categorized into a high-risk depression group and 24 participants serve as a low-risk control group. All participants were assessed using the PROMIS (Patient-Reported Outcomes Measurement Information System) Depression Scale to ensure the reliability and consistency of depression evaluation. EEG signals were collected using 62 electrodes mounted on the scalp of each participant (as illustrated in Figure 1), with a sampling frequency of 500 Hz. Signal quality was ensured through standardized electrode placement.

During the data acquisition process, EEG signals not only reflect brain activity but are also susceptible to environmental noise and physiological artifacts. Therefore, a series of preprocessing steps is required to extract discriminative features from the raw signals [45]. The primary preprocessing step is filtering, which aims to eliminate low-frequency drift and high-frequency noise [46]. In this study, a band-pass filter with a frequency range of 0.5‐50 Hz was applied to the EEG signals. The filtered signals are able to better capture meaningful neural activity while effectively suppressing most physiological and instrumental noise components [47].

EEG signals are sensitive to external factors such as reaction time variability and participant fatigue, and thus a fixed-duration segment is typically selected for analysis [48]. In this work, 60 s of continuous EEG data were extracted from each participant for subsequent analysis. While increasing the amount of data can enhance model robustness, the limited number of participants makes data augmentation a crucial step [49]. Accordingly, a slicing strategy was used to segment the EEG signals into shorter time series, with each segment having a duration of 1 s. Through this process, the original EEG recordings were transformed into multiple short-term sequences, effectively enlarging the dataset and improving the learning performance of the proposed models.

**Figure 1.**
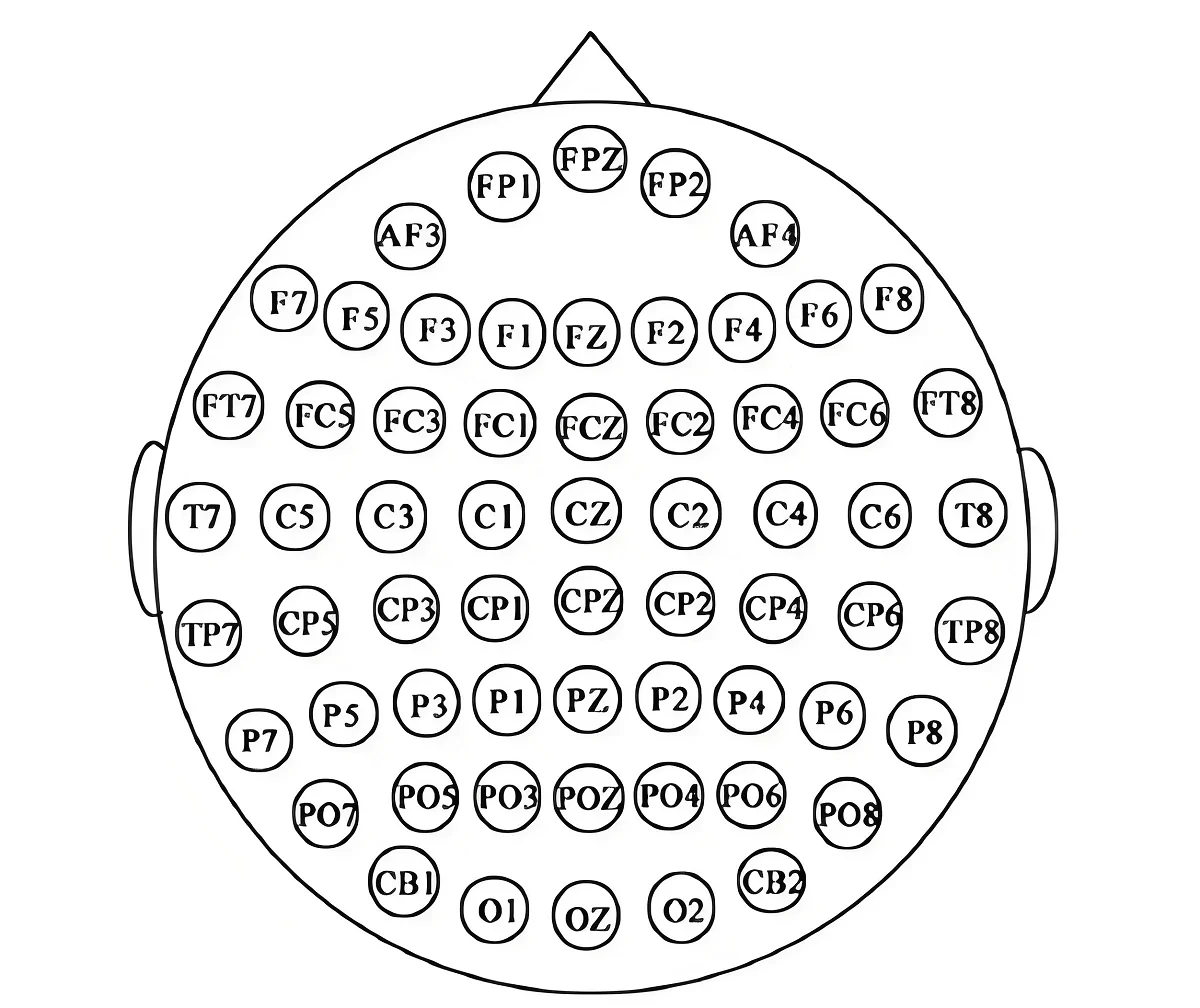
Electrode diagram.

#### EEG Signal Preprocessing

Frequency-domain analysis of EEG signals plays an important role in depression recognition, as EEG activities in different frequency bands reflect distinct cognitive states of the brain [45]. Common EEG frequency bands include delta (δ, 0.5‐4 Hz), theta (θ, 4‐8 Hz), alpha (α, 8‐13 Hz), beta (β, 13‐30 Hz), and gamma (γ, above 30 Hz), with each band corresponding to different physiological and psychological activities [50]. In EEG signals of patients with depression, abnormal variations are typically observed across multiple frequency bands, particularly in the θ, α, β, and δ bands [51]. Frequency-domain analysis enables effective characterization of these variations and provides informative features for subsequent depression recognition.

The selection of θ, α, β, and δ bands is motivated by their close association with depression-related neural activity. The δ band is related to slow-wave cortical activity and may reflect abnormal low-frequency neural regulation. The θ band is associated with cognitive control, emotional processing, and fronto-limbic regulation, which are frequently affected in depressive states. The α band is commonly linked to resting-state cortical activation and affective regulation, and abnormal α activity or asymmetry has been widely investigated in depression-related EEG studies. The β band reflects relatively active cognitive and emotional states and may capture changes in arousal and neural excitability. Therefore, band-power features extracted from these frequency ranges can provide compact and physiologically meaningful representations of depression-related EEG activity. Compared with raw EEG signals, PSD-based band-power features reduce temporal redundancy while preserving discriminative spectral information, making them suitable for subsequent nonlinear feature modeling using KAN-based architectures.

PSD is an important tool for characterizing the distribution of signal power across different frequency components [52,53]. In this study, PSD analysis was adopted to extract frequency-domain features from EEG signals. The general form of PSD can be expressed as:


(1) $$P_{xx}(f)=\underset{T\to \infty }{\lim}\frac{1}{T}|X_{T}(f){|}^{2}$$

where $P_{xx}(f)$ denotes the PSD at frequency f,$X_{T}(f)$ represents the Fourier transform of the EEG signal within duration T, and T is the signal duration.

In practical implementation, the Welch method was used to obtain a stable PSD estimate. The Welch method can be regarded as an averaged periodogram-based PSD estimation approach. Specifically, each 1 s EEG segment was first divided into shorter overlapping subsegments. A window function was then applied to each subsegment to reduce spectral leakage. Subsequently, the periodogram of each windowed subsegment was calculated, and the final PSD estimate was obtained by averaging all periodograms:


(2) $${\hat{P}}_{xx}\left(f\right)=\frac{1}{L}\sum_{l=1}^{L}P_{l}\left(f\right)$$

where $P_{l}(f)$ denotes the periodogram of the l-th windowed subsegment, and L is the number of subsegments. In this study, the Welch-based PSD estimation was implemented using the scipy.signal.welch function.

Based on the estimated PSD, the band-power features of the θ, α, β, and δ frequency bands were extracted by integrating the PSD values within each frequency range:


3) $$BP_{b}=\int_{f_{1}}^{f_{2}}{\hat{P}}_{xx}\left(f\right)df$$

where $BP_{b}$ denotes the power of a specific frequency band b, and $f_{1}$ and $f_{2}$ represent the lower and upper frequency bounds of that band. These band-power features were then used as frequency-domain inputs for subsequent depression recognition models.

To ensure comparability of features across different EEG channels, the extracted PSD features are normalized. Z-score normalization is adopted, which is defined as follows:


(4) $$z=\frac{x-\mu }{\sigma }$$

where x denotes the feature value, μ and σ represent the mean and SD of the feature, respectively, and z denotes the normalized feature. The normalized features are then used as inputs to the subsequent depression recognition models.

In addition, ANOVA [54] is conducted after feature extraction to evaluate the differences in PSD features between the depression group and the control group across different frequency bands. The ANOVA results indicate that significant differences exist between the 2 groups in the θ, α, β, and δ bands, which further confirms the effectiveness of frequency-domain features in depression recognition.

Through the above processing pipeline, frequency-domain features of EEG signals are successfully extracted, providing a foundation for subsequent depression recognition. Classification methods based on these frequency-domain features can improve the accuracy of depression recognition and demonstrate potential practical value.

### KAN-Based Depression Recognition Model Construction

#### CNN Model

CNNs are a class of deep learning models widely used in image recognition, speech processing, and EEG signal analysis [55]. Their main advantage lies in the ability to automatically extract features by learning local patterns through convolution operations, while pooling layers are used to reduce computational complexity and improve generalization performance [56].

In EEG signal classification, CNNs can effectively capture spatiotemporal features and enhance classification accuracy [57]. A typical CNN architecture consists of an input layer, convolutional layers, pooling layers, and fully connected layers [58]. The input layer receives the preprocessed EEG data. The convolutional layers extract local features using sliding convolutional kernels and use the ReLU activation function to enhance nonlinear representation capability [59]. The convolution operation is defined as follows:


(5) $$Y_{ij}=\sum_{m=1}^{k}\sum_{n=1}^{k}X_{i+m,j+n}W_{m,n}+b$$

where X denotes the input signal, W represents the convolution kernel, b is the bias term, and Y is the resulting feature map after convolution.

The pooling layer is used for dimensionality reduction, decreasing computational cost and preventing overfitting. A commonly used pooling strategy is max pooling, which is defined as:


(6) $$Y_{ij}=\max(x_{i,j},x_{i+1},x_{i,j+1},x_{i+1,j+1})$$

where x denotes the input feature map and Y represents the pooled feature map.

Finally, the fully connected layer maps the extracted features to the corresponding class labels, and the Softmax function is applied to compute class probabilities, thereby completing the depression recognition task. The computation of the fully connected layer is expressed as:


(7) $$y=f(W_{x}+b)$$

where x denotes the output of the previous layer, W is the weight matrix, b represents the bias term, and f denotes the activation function, which is typically ReLU or Sigmoid.

#### KAN Model

KAN is a deep neural network architecture based on Kolmogorov-Arnold theory and is well suited for complex nonlinear mapping problems, particularly in tasks involving time-series data and high-dimensional feature learning. The core idea of KAN is to enhance model representation capability through multilayer nonlinear mappings, making it especially suitable for complex pattern recognition tasks such as EEG signal analysis.

The structure of the KAN model can be more intuitively understood through a schematic illustration, as shown in Figure 2.

In EEG signal analysis, KAN is capable of extracting deep features and can be applied to classification and disease diagnosis tasks. Although KAN has not yet been directly applied to depression recognition, its strong feature extraction and modeling capabilities provide theoretical support for EEG signal pattern analysis [60]. A KAN model is composed of multiple mapping units (KAN units), where each layer constructs a high-dimensional feature space through nonlinear transformations to explore deep patterns in the data [61].

**Figure 2.**
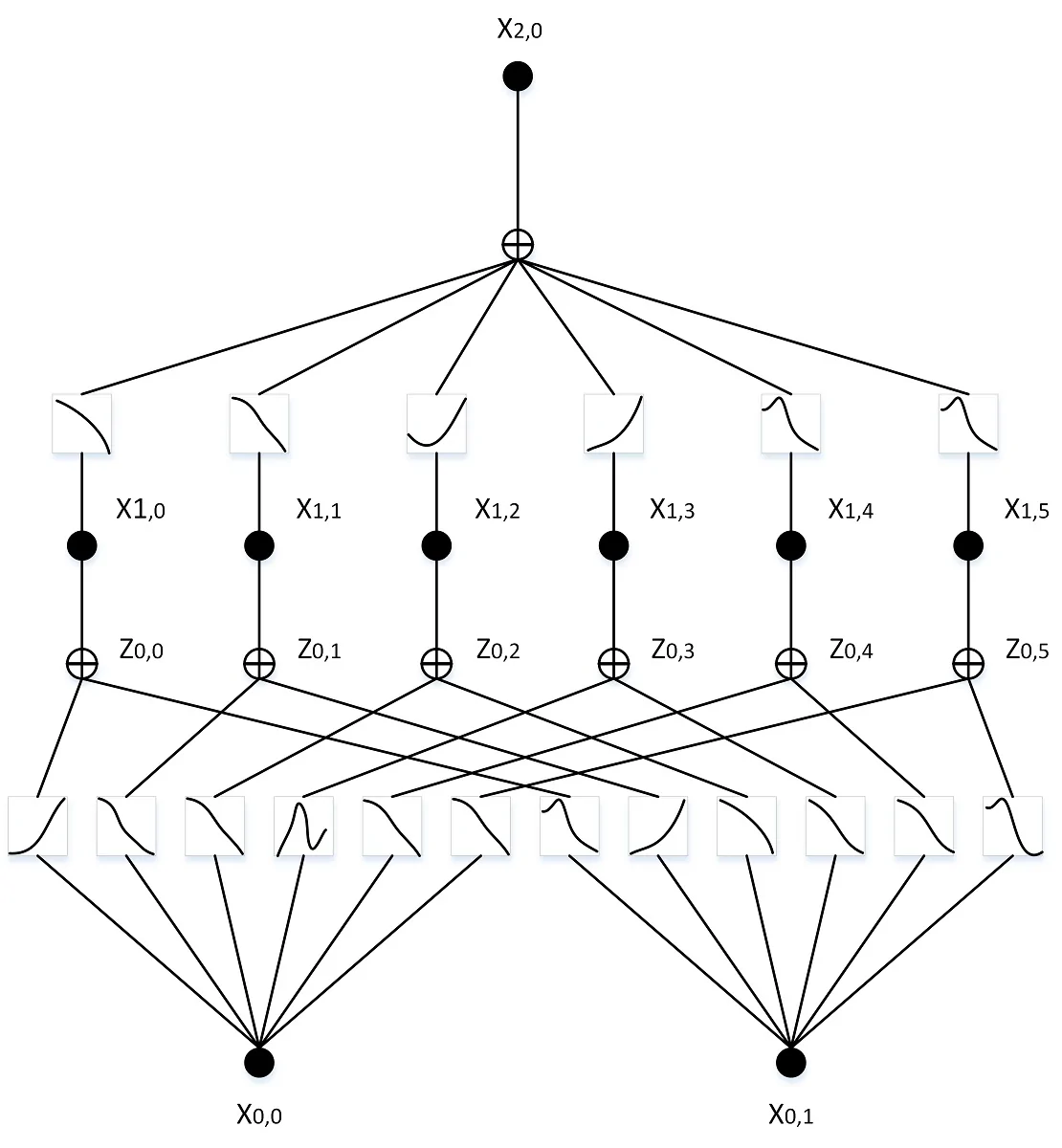
Schematic diagram of the KAN model architecture. KAN: Kolmogorov-Arnold Networks.

The core architecture of a KAN model generally consists of the following components:

- Regarding the input layer, the number of input nodes is equal to the data dimensionality. In EEG signal processing, each node corresponds to feature data from an EEG channel.
- Regarding the KAN units (Kolmogorov-Arnold units), these are the core computational units of the model, consisting of multiple parameters and nonlinear activation functions that perform nonlinear mappings on the input. The mathematical formulation is given as follows:

(8) $$y_{i}=f(\sum_{j=1}^{n}w_{ij}x_{j}+b_{j})$$

where $x_{j}$ denotes the j−th input feature, $w_{ij}$ represents the weight between this feature and the i−th KAN unit, $b_{j}$ is the bias term, and f denotes the nonlinear activation function. Common activation functions include ReLU, Sigmoid, and Tanh.

- Regarding the hidden layers, each hidden layer is composed of multiple KAN units, and the output of 1 layer serves as the input to the next. Through successive nonlinear mappings, increasingly deeper features are learned.
- Regarding the output layer, a fully connected layer maps the learned features to the final class labels, and the Softmax activation function is typically used to compute class probabilities.

The main advantage of the KAN model lies in its strong nonlinear mapping capability, which enables effective capture of complex patterns in EEG signals and provides high-precision feature representations for pattern recognition and classification tasks.

#### ConvKAN Model

#### Overview

The ConvKAN model integrates CNN and KAN by incorporating KAN into convolutional layers, enabling enhanced nonlinear feature transformation. The core idea of this model is to enhance the expressive capability of convolutional layers by incorporating the nonlinear feature mapping of KAN on the basis of traditional convolution kernels. This approach is particularly suitable for handling high-dimensional and complex data, such as EEG signals. The key innovation of ConvKAN lies in replacing traditional convolution kernels with KANLinear layers, enabling convolution operations to learn more complex feature transformations.

#### KANLinear Layer Design

The KANLinear layer is the core module of ConvKAN. It uses B-spline interpolation to perform nonlinear transformations on input features. In traditional convolutional layers, local features are extracted through dot-product operations between convolution kernels and input features. In contrast, KANLinear performs feature transformation through an interpolation mechanism. Assuming the input feature is denoted as $x\in R^{d}$, the output $y\in R^{m}$ of the KANLinear layer can be expressed as:


(9) y=W·x+b

where $W\in R^{m\times d}$ denotes the weight matrix and $b\in R^{m}$ represents the bias term. The KANLinear layer transforms the input features x through the interpolation function to generate a new feature representation y.

#### ConvKAN Convolutional Layer Design

The ConvKAN convolutional layer combines traditional convolution operations with the KANLinear layer to form a new type of convolution. While conventional convolution extracts local features by sliding convolution kernels, ConvKAN replaces the convolution kernel with a KANLinear layer, allowing it to capture more complex nonlinear relationships in the input data. The workflow of ConvKAN is as follows:

Regarding the unfold operation, first, the input data $X\in R^{B\times C\times H\times W}$ (where B denotes the batch size, C is the number of channels, and H and W represent the height and width of the input image, respectively) are unfolded into a matrix $X_{unf}$, where each column corresponds to the features of a local region involved in the convolution operation. The unfolding operation is defined as:


(10) $$X_{unf}=Unfold(X)$$

Regarding the KAN processing, the unfolded matrix $X_{unf}$ is then fed into the KANLinear layer for feature transformation. This process effectively captures complex nonlinear relationships in the input features. The output $Y\in R^{B\times G\times O}$ of the KANLinear layer can be expressed as:


(11) $$Y=KANLinear(X_{unf})$$

where G indicates the number of groups and O represents the dimensionality of the output features. In this way, the KANLinear layer can convert the input features $X_{unf}$ into outputs Y with nonlinear characteristics.

Regarding the output reshaping, the output features Y produced by the KANLinear layer are reshaped into the standard output format of a convolution operation. Specifically, the output features are rearranged according to the predefined output shape, resulting in the final convolution output $Y_{out}\in R^{B\times C^{\prime }\times H^{\prime }\times W^{\prime }}$, where $C^{`}$ denotes the number of output channels, and $H^{`}$ and $W^{`}$ represent the height and width of the output image, respectively.


(12) $$Y_{out}=\text{Reshape(Y)}$$

In ConvKAN, the spatial dimensions of the output (height $H_{out}$ and width $W_{out}$) are determined based on the input size and convolution kernel parameters. Assuming the input height and width are $H_{in}$ and $W_{in}$, respectively, the kernel size is $K_{h}\times K_{w}$, the stride is $S_{h}\times S_{w}$, and the padding is$P_{h}\times P_{w}$, the output height and width can be calculated as follows:


(13) $$\begin{array}{c} H_{out}=\left\lfloor \frac{H_{in}+2P_{h}-K_{h}}{S_{h}}\right\rfloor +1\\ W_{out}=\left\lfloor \frac{W_{in}+2P_{w}-K_{w}}{S_{w}}\right\rfloor +1 \end{array}$$

where $\left\lfloor \cdot \right\rfloor$ denotes the floor operation. Through this formulation, ConvKAN ensures that the output dimensions of the convolution operation meet the expected requirements.

The specific model architecture is illustrated in Figure 3.

**Figure 3.**
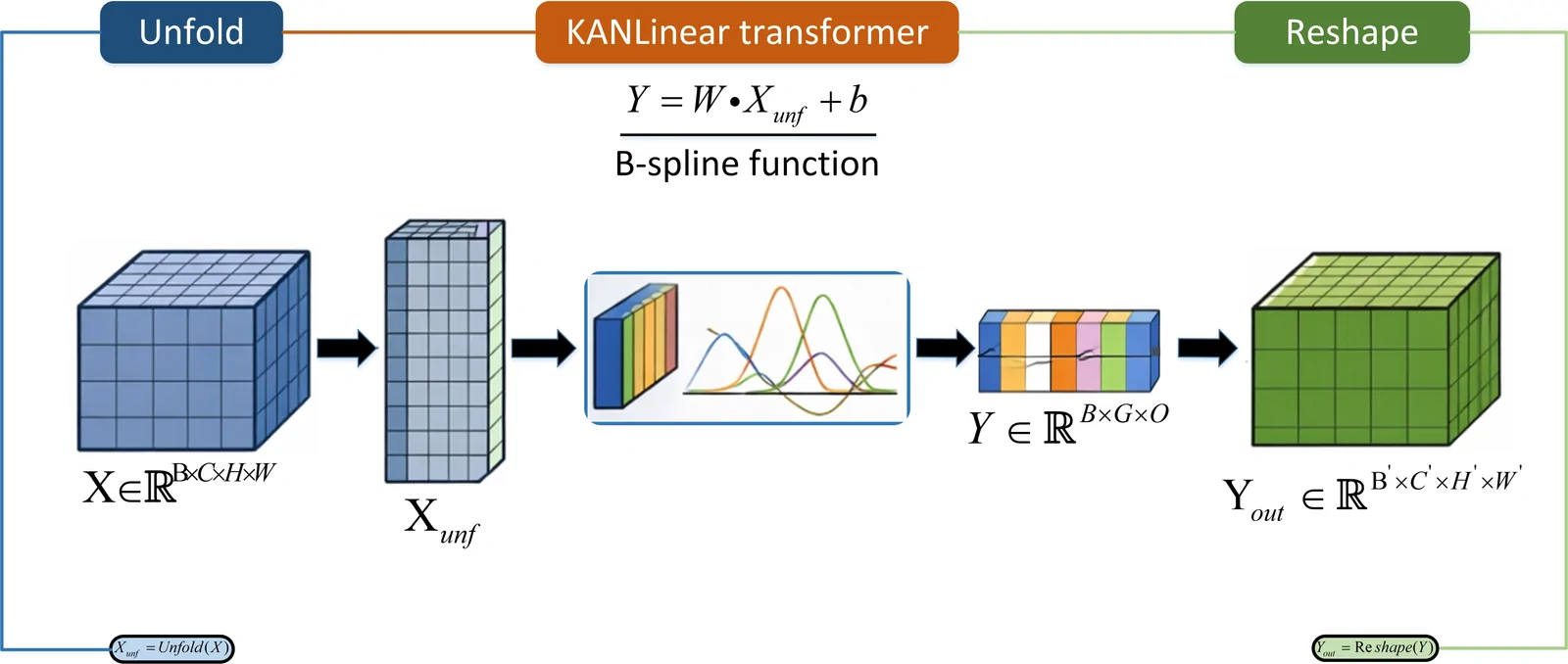
Architecture of the ConvKAN model.

Based on traditional CNNs, the ConvKAN model effectively enhances the modeling capability for complex data by introducing the KANLinear layer. In particular, when processing time-series data such as EEG signals, ConvKAN is able to capture high-dimensional features that cannot be expressed by conventional convolution operations.

### ConvKAN Efficient Model

ConvKAN Efficient is an efficiency-optimized variant of ConvKAN designed to improve the computational process and training stability of KAN-based convolution. It primarily enhances computational efficiency and model performance through mechanisms such as dynamic grid updating, optimized weight initialization, and improved regularization, showing significant advantages especially in processing EEG data. The model architecture is illustrated in Figure 4.

**Figure 4.**
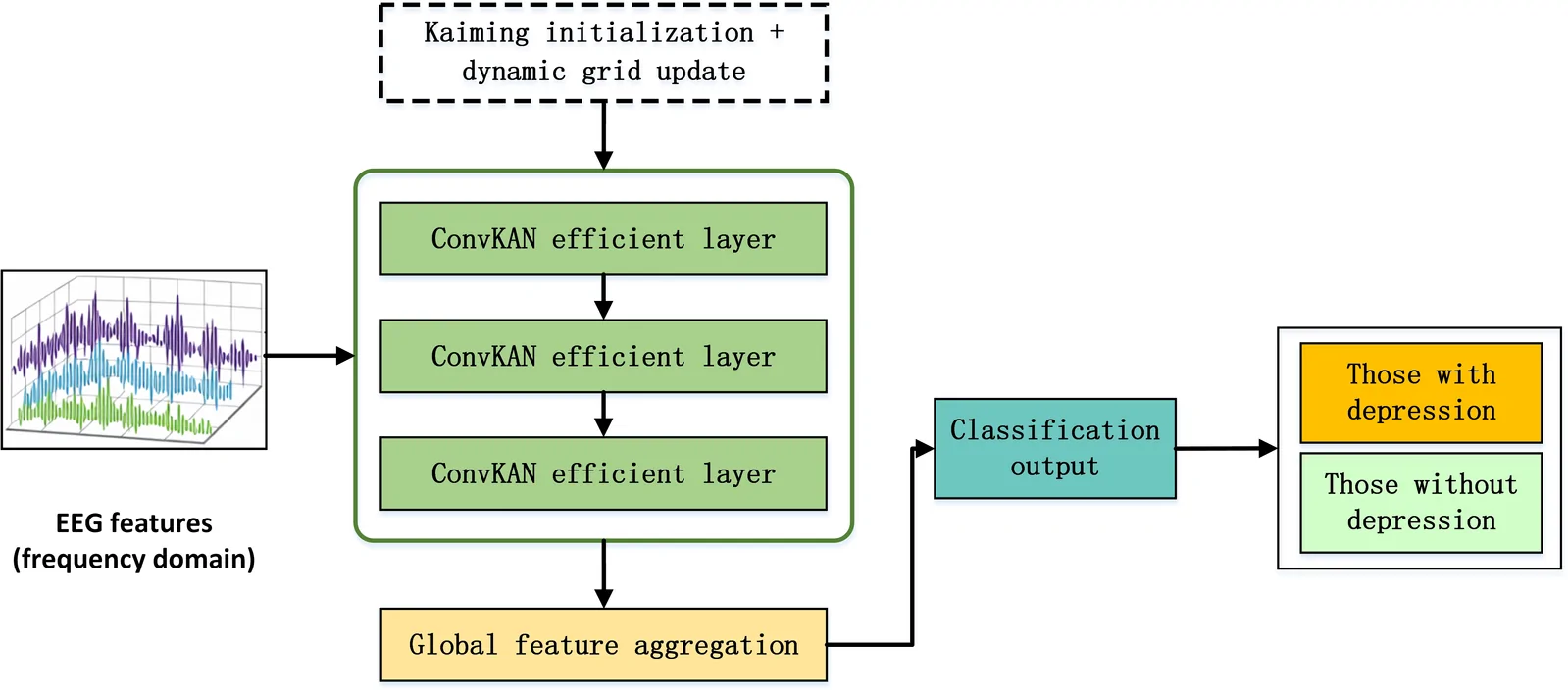
Architecture of the ConvKAN Efficient model. EEG: electroencephalogram.

Regarding the dynamic grid updating mechanism, the dynamic grid updating mechanism is introduced to enhance the adaptability of spline-based nonlinear mappings in ConvKAN Efficient. In KAN-based convolution, the nonlinear transformation is represented by learnable spline functions defined on a set of grid points. If the grid remains fixed during training, the spline basis may not sufficiently adapt to the distributional variations of EEG features across channels and frequency bands. Therefore, ConvKAN Efficient updates the grid according to the empirical distribution of input features, allowing the spline basis functions to better fit the feature space.

Let the unfolded local feature patches generated by the convolution operation be denoted as:


(14) $$Z\in R^{N_{p}\times d}$$

where $N_{p}$ denotes the number of local patches and d represents the input dimensionality of the KANLinear layer. For the j-th feature dimension, the initial grid is uniformly constructed as:


(15) $$g_{i,j}^{0}=x_{j}^{min}+i\cdot \frac{x_{j}^{max}-x_{j}^{min}}{G},i=0,1,...,G-x_{j}^{min}$$

where G is the number of grid intervals, and $x_{j}^{\min}$ and $x_{j}^{\max}$ denote the minimum and maximum values of the corresponding feature dimension.

During training, the adaptive grid is estimated from the empirical distribution of the current input batch. Specifically, the adaptive grid points are obtained through quantile-based estimation: $g_{i,j}^{ada}=Q_{j}(\frac{i}{G}),i=0,1,...,G$ (16)

where $GQ_{j}(\cdot )$ denotes the empirical quantile function of the j-th feature dimension. This strategy allows more grid points to be assigned to feature-dense regions, thereby improving the local approximation capability of the spline functions.

To avoid excessive fluctuations caused by mini-batch variation, the adaptive grid is combined with a uniformly spaced grid:


(17) $$g_{i,j}^{new}=\varepsilon g_{i,j}^{uni}+(1-\varepsilon )g_{i,j}^{ada}$$

where $g_{i,j}^{uni}$ denotes the uniform grid estimated from the current batch range, and ε is a blending coefficient used to balance numerical stability and data adaptivity.

After the grid is updated, the spline-based nonlinear transformation can be expressed as:


(18) $$\phi \left(x\right)=w_{b}b\left(x\right)+\sum_{l}w_{s,l}B_{l}\left(x;G^{new}\right)$$

where $w_{b}$ denotes the base weight, b(x) is the base activation function, $w_{s,l}$ represents the spline coefficient, and $B_{l}(x;G^{new})$ denotes the l-th B-spline basis function defined on the updated grid.

Regarding the optimized weight initialization, for weight initialization, Kaiming initialization (He initialization) is used. This method considers the input and output dimensions of each layer and initializes weights according to the following formula:


(19) $$W_{o}=N(0,\sqrt{2/fan_{in}})$$

where $N(0,\sqrt{2/fan_{in}})$ denotes a Gaussian distribution and $fan_{in}$ is the number of input neurons in the current layer. With Kaiming initialization, ConvKAN Efficient achieves better gradient propagation in the early stages of training, improving training stability, especially in deep networks.

Regarding the improved regularization mechanism, to prevent overfitting and enhance generalization, ConvKAN Efficient incorporates an improved regularization mechanism. For the weights W of the spline basis functions, L1 regularization is applied to constrain model complexity:


(20) $$L_{reg}=\lambda \sum_{i}\left|W_{i}\right|$$

where λ is the regularization hyperparameter and $W_{i}$ represents the parameters of each spline basis function weight in the model. L1 regularization forces the network to learn sparse convolution kernels, thereby controlling complexity while maintaining efficiency. Additionally, ConvKAN Efficient incorporates entropy regularization to further enhance model stability and robustness:


(21) $$L_{entropy}=-\sum_{i}p_{i}\log(p_{i})$$

where $p_{i}$ denotes the normalized probability distribution of each spline basis function weight.

In the regularization mechanism of ConvKAN Efficient, L1 regularization and entropy regularization play different but complementary roles. L1 regularization is imposed on the spline coefficients to encourage sparsity in the learned nonlinear mapping functions. By suppressing redundant spline coefficients, L1 regularization helps reduce unnecessary basis responses and alleviates overfitting, which is particularly important for EEG datasets with limited sample sizes.

Entropy regularization further constrains the distribution of normalized spline weights. Its purpose is to improve the stability of spline-basis usage during training and prevent the nonlinear representation from being dominated by a small number of basis functions. By regulating the information distribution among spline components, entropy regularization enhances the robustness and generalization capability of ConvKAN Efficient. Therefore, L1 regularization mainly contributes to sparsity and complexity control, whereas entropy regularization improves distributional stability and robustness. Together, these 2 regularization terms help ConvKAN Efficient learn more stable and discriminative nonlinear EEG feature representations.

Regarding the improved computational efficiency, ConvKAN Efficient leverages the optimized dynamic grid updating mechanism and efficient convolution kernel computation to achieve higher computational efficiency compared with ConvKAN. During actual training, ConvKAN Efficient can complete training in a shorter time while maintaining high classification performance, which is particularly advantageous when handling large-scale EEG data.

ConvKAN Efficient builds upon ConvKAN by introducing dynamic grid updating, optimized weight initialization, and stronger regularization, providing comprehensive improvements in feature extraction, training stability, and computational efficiency.

### Experimental Setup

#### Evaluation Metrics

To comprehensively assess the performance of the KAN-based models, several commonly used classification evaluation metrics were used, including accuracy, precision, recall, and *F*_1_-score. These metrics reflect the model’s performance from different perspectives and are particularly useful for providing a more complete evaluation when handling imbalanced datasets.

Accuracy is calculated as:


(22) $$Accuracy=\frac{TP+TN}{TP+TN+FP+FN}$$

where TP, TN, FP, and FN represent true positives, true negatives, false positives, and false negatives, respectively.

Precision and recall are computed as:


(23) $$Precision=\frac{TP}{TP+FP},Recall=\frac{TP}{TP+FN}$$

The *F*_1_-score is defined as:


(24) $$F1=2\cdot \frac{Precision\cdot Recall}{Precision+Recall}$$

By using these metrics, the model’s performance in depression recognition tasks can be comprehensively evaluated, ensuring reliable classification capability across different scenarios.

In addition to the above classification metrics, statistical significance analysis was further conducted to examine whether the performance differences between ConvKAN Efficient and the baseline deep learning models were statistically meaningful. Since the evaluated models were tested on the same held-out test set, the McNemar exact test was adopted based on paired prediction outcomes.

For 2 classifiers M1 and M2, the McNemar test focuses on the discordant prediction pairs. Let $n_{01}$ denote the number of samples incorrectly classified by M1 but correctly classified by M2, and $n_{10}$ denote the number of samples correctly classified by M1 but incorrectly classified by M2. The test evaluates whether the 2 discordant counts are significantly different. In this study, ConvKAN Efficient was compared with CNN and ConvKAN, respectively. A *P* value less than .05 was considered statistically significant.

#### Comparison Methods

Three classical machine learning methods—linear discriminant analysis (LDA), DT, and LR—and 3 deep learning models—CNN, ConvKAN, and ConvKAN Efficient—were used for comparison experiments to evaluate their performance in depression recognition.

LDA classifies data by finding the optimal projection direction. It offers high computational efficiency but is limited by the linearity assumption, which reduces its effectiveness on complex EEG data. DT performs classification based on feature splits, providing an interpretable decision process but is prone to overfitting, especially in small-sample scenarios. LR is a linear classification model suitable for binary tasks; it is computationally simple but performs poorly on nonlinear data.

CNN automatically extracts local features from EEG signals via convolutional layers and performs classification using fully connected layers. Its advantage lies in strong feature learning capability, but the fixed convolution kernels may restrict its modeling of complex nonlinear signals. ConvKAN extends CNN by integrating KAN, allowing the convolution kernels to adaptively adjust, thereby extracting EEG features more accurately and improving depression recognition performance, particularly for small-sample datasets. ConvKAN Efficient further enhances model stability and feature representation while maintaining computational efficiency through optimized weight initialization and improved regularization mechanisms.

#### Parameter Settings

The experiments were conducted on a hardware platform equipped with a 12th Gen Intel Core i7-12700H 2.30 GHz processor, 16 GB DDR4 RAM, and 512 GB SSD storage, running Windows 11. The development environment was PyCharm, with Python (version 3.8; Python Software Foundation) as the programming language and PyTorch 2.4.1 as the deep learning framework, supporting GPU acceleration. The dataset was split into a training set containing 2400 samples and a test set containing 600 samples. To avoid data leakage, the dataset was divided in a participant-independent manner, ensuring that EEG segments from the same participant did not simultaneously appear in both the training and test sets.

For the 3 deep learning models used for depression recognition (traditional CNN, ConvKAN, and ConvKAN Efficient), the cross-entropy loss function (CrossEntropyLoss) was used for multiclass classification. The Adam optimizer was used with a learning rate of 0.0001 and a batch size of 16. The models were trained for 100 epochs, with loss and accuracy calculated during training, and the final model performance evaluated on the test set.

#### Ethical Considerations

This study involved a secondary analysis of the publicly available EDRA dataset. No new EEG data were collected from human participants, and the authors did not have any direct interaction with participants. The dataset was anonymized before public release, and no personally identifiable information was accessible during the analysis.

The original data collection and experimental procedures were conducted by the dataset providers, who obtained ethical approval and informed consent from all participants according to their institutional requirements. Since the present study only involved the analysis of an existing anonymized dataset and did not involve the collection of new human participant data, additional institutional review board approval was not required for this secondary analysis.

## Results

### Metric Evaluation

#### Overview

In this study, 3 traditional machine learning models (DT, LR, and LDA) and 3 deep learning models (CNN, ConvKAN, and ConvKAN Efficient) were used to classify EEG signals for depression recognition. Model performance was evaluated using accuracy, precision, recall, and *F*_1_-score. The experimental results are summarized in Table 1.

**Table 1.** Comparison of depression recognition results across different models.

Model	Accuracy (%)	Precision (%)	Recall (%)	*F*_1_-score (%)
LDA^a^	88.33	88.38	88.33	88.32
DT^b^	89.50	89.63	89.50	89.48
LR^c^	90.00	90.01	90.00	90.00
CNN^d^	89.00	89.02	89.00	88.99
ConvKAN	90.50	90.97	90.50	90.46
ConvKAN Efficient	94.83	94.97	94.83	94.83

a LDA: linear discriminant analysis.

b DT: decision tree.

c LR: logistic regression.

d CNN: convolutional neural network.

#### Performance of Traditional Machine Learning Models

The traditional machine learning models achieved relatively competitive performance in EEG-based depression recognition, demonstrating that handcrafted frequency-domain features still contain substantial discriminative information. Among these models, LR achieved the highest classification accuracy of 90%, outperforming DT (89.50%) and LDA (88.33%). The precision and *F*_1_-score results also indicate that LR exhibited relatively balanced classification capability, suggesting that linear decision boundaries can still capture certain global statistical characteristics of EEG spectral features.

LDA achieved the lowest overall performance among the 3 conventional methods. This phenomenon may mainly result from its strong statistical assumptions regarding feature distributions. Specifically, LDA assumes that different classes follow Gaussian distributions with identical covariance matrices. However, EEG signals are highly nonlinear, nonstationary, and participant-specific, making it difficult for these assumptions to hold in practical depression recognition tasks. Consequently, LDA showed weaker adaptability to the complex distributions of EEG features, leading to reduced classification performance.

The DT model achieved slightly better results than LDA, indicating that tree-based nonlinear partitioning can partially capture complex EEG feature relationships. Since DT constructs hierarchical decision boundaries through recursive feature splitting, it provides strong interpretability and local feature discrimination capability. Nevertheless, DT is highly sensitive to training data variations and may suffer from overfitting, particularly in relatively small EEG datasets. This limitation may explain why DT failed to further improve classification accuracy despite its nonlinear modeling capability.

LR demonstrated comparatively stable performance across all evaluation metrics. Although LR is essentially a linear classifier, its probabilistic decision mechanism enables effective modeling of global trends in EEG feature distributions. Moreover, after PSD extraction and *z* score normalization, the EEG features exhibit improved linear separability, which further benefits LR classification performance. The relatively competitive results of LR also suggest that frequency-domain EEG features possess meaningful statistical regularities associated with depression-related neural activity.

The results indicate that conventional machine learning approaches can achieve acceptable performance when appropriate EEG preprocessing and feature extraction strategies are used. However, their limited nonlinear representation capability restricts their ability to fully model the complex spatiotemporal dependencies and dynamic neural patterns present in EEG signals.

#### Performance of Deep Learning Models

Compared with traditional machine learning approaches, deep learning models demonstrated stronger nonlinear modeling capability and improved feature learning performance. However, the experimental results also reveal that not all deep learning architectures are equally effective for EEG-based depression recognition.

The conventional CNN achieved an accuracy of 89%, which was slightly lower than LR and DT. This result suggests that standard CNN architectures do not necessarily provide significant advantages for EEG signal classification tasks. Unlike image data, EEG signals contain strong temporal dynamics, nonlinear oscillatory patterns, and cross-channel dependencies. Traditional CNNs mainly focus on extracting local spatial features through fixed convolution kernels, which may limit their ability to effectively capture long-range temporal dependencies and adaptive nonlinear relationships within EEG signals. In addition, CNNs generally require large-scale datasets to fully exploit their representation capability, whereas EEG depression datasets are often relatively limited in size, increasing the risk of insufficient feature learning and overfitting.

ConvKAN achieved notable performance improvement over CNN by integrating the KAN adaptive nonlinear mapping mechanism into convolutional operations. Specifically, ConvKAN improved the classification accuracy from 89% to 90.50%, while also achieving higher precision (90.97%) and *F*_1_-score (90.46%). These improvements indicate that KAN-based nonlinear transformations can effectively enhance EEG feature representation capability.

The superior performance of ConvKAN can be attributed to several important factors. First, the KANLinear layer replaces traditional fixed linear convolution kernels with adaptive nonlinear mappings, enabling the model to learn more flexible feature transformation functions. This characteristic is particularly beneficial for EEG signals, whose neural oscillations often exhibit highly nonlinear and nonstationary behaviors. Second, the spline-based interpolation mechanism in KAN allows the model to better approximate complex feature distributions while preserving local signal structures. Third, adaptive feature transformation enhances the model’s ability to capture subtle depression-related neural variations across different EEG frequency bands.

Building upon ConvKAN, ConvKAN Efficient further improved model performance through multiple optimization strategies, including dynamic grid updating, optimized weight initialization, and enhanced regularization mechanisms. Experimental results demonstrate that ConvKAN Efficient achieved the best overall classification performance, with accuracy, precision, recall, and *F*_1_-score all exceeding 94%.

The significant performance improvement of ConvKAN Efficient indicates that the proposed optimization strategies effectively enhance both feature learning capability and model generalization performance. The dynamic grid updating mechanism allows the model to adaptively adjust spline representations according to the data distribution during training, thereby improving nonlinear approximation capability. Meanwhile, Kaiming initialization improves gradient propagation stability in deep architectures, reducing the risk of vanishing or exploding gradients during training. Furthermore, the incorporation of L1 regularization and entropy regularization helps constrain model complexity and suppress overfitting, which is particularly important for EEG datasets with limited sample sizes.

Another important observation is that ConvKAN Efficient achieved simultaneously high precision and recall. High precision indicates that the model effectively reduces false positive predictions, while high recall demonstrates strong sensitivity in identifying depression-related EEG patterns. In practical clinical applications, maintaining both high precision and recall is crucial because missed diagnoses and false alarms may both negatively affect clinical decision-making. Therefore, the balanced performance achieved by ConvKAN Efficient highlights its potential value for computer-aided depression screening systems.

#### Comparative Analysis

Overall, the experimental results demonstrate a clear performance hierarchy among the evaluated models. Traditional machine learning approaches achieved acceptable classification accuracy, indicating that PSD-based frequency-domain features contain meaningful depression-related information. However, their limited nonlinear representation capability restricts their ability to fully capture the complex neural dynamics embedded in EEG signals.

Although CNNs are widely successful in image analysis tasks, their performance advantage was not fully reflected in this EEG classification task. This finding further emphasizes that EEG signals differ substantially from image data in terms of statistical properties and temporal dynamics. Fixed convolution kernels may be insufficient for modeling the highly nonlinear and participant-dependent characteristics of EEG signals.

In contrast, the introduction of KAN significantly improved EEG feature learning capability. ConvKAN demonstrated that adaptive nonlinear mappings can effectively enhance the representation of EEG features, leading to improved classification performance. More importantly, ConvKAN Efficient further strengthened this advantage through optimization strategies that improved training stability, feature adaptability, and model generalization.

From a broader perspective, the experimental findings suggest that KAN-based architectures provide a promising direction for EEG signal analysis. Unlike traditional CNNs that rely heavily on fixed linear convolution operations, KAN-based models offer more flexible nonlinear function approximation capability, making them particularly suitable for complex biomedical signals. The investigated ConvKAN Efficient model not only achieved superior classification performance but also maintained computational efficiency through architectural optimization, which is important for future real-time and wearable EEG applications.

To further verify whether the performance improvements of ConvKAN Efficient were statistically significant, a McNemar exact test was conducted based on the paired prediction results of the same test set. The results are summarized in Table 2.

**Table 2.** Statistical comparison of model performance using McNemar exact test.

Model1	Model2	both_correct	both_wrong	model_1_correct_model_2_wrong	model_1_wrong_model_2_correct	discordant_pairs
ConvKAN_Efficient	CNN^a^	521	20	44	15	59
ConvKAN_Efficient	ConvKAN	544	17	21	18	39

a CNN: convolutional neural network.

As shown in Table 2, when ConvKAN Efficient was compared with CNN, 44 samples were correctly classified only by ConvKAN Efficient, whereas 15 samples were correctly classified only by CNN. The corresponding *P* value was .000204, indicating a statistically significant difference between the 2 models. When ConvKAN Efficient was compared with ConvKAN, 21 samples were correctly classified only by ConvKAN Efficient, whereas 18 samples were correctly classified only by ConvKAN. The corresponding *P* value was .7493, indicating that the difference in paired prediction outcomes did not reach statistical significance. Nevertheless, ConvKAN Efficient achieved higher overall performance than ConvKAN across the main evaluation metrics, suggesting a potential performance benefit of the optimization strategies.

These results demonstrate that the performance improvement of ConvKAN Efficient is not only reflected in overall classification metrics but also statistically supported by paired prediction analysis. Therefore, ConvKAN Efficient provides a more reliable classification performance than CNN and standard ConvKAN under the same experimental setting.

### Computational Efficiency Analysis

To further examine the computational characteristics of the evaluated deep learning models, CNN, ConvKAN, and ConvKAN Efficient were compared in terms of trainable parameters, model size, training time per epoch, and single-sample inference time. In addition to classification accuracy, these indicators provide a more direct assessment of model complexity and computational cost.

The comparison results are shown in Table 3.

**Table 3.** Computational efficiency comparison of deep learning models.

Model	Accuracy (%)	Trainable parameters	Model size (MB)	Training time/epoch (s)	Inference time/sample (s)
CNN^a^	89.00	265,346	1.01	1.5511	0.000161
ConvKAN	90.50	433,730	1.65	2.5017	0.003761
ConvKAN Efficient	94.83	433,730	1.65	2.1972	0.002600

a CNN: convolutional neural network.

As shown in Table 3, CNN has the lowest computational cost due to its standard convolutional structure. However, its classification accuracy is lower than that of the KAN-based models, indicating that conventional convolution may be insufficient for capturing complex nonlinear EEG feature distributions. Compared with CNN, ConvKAN introduces spline-based nonlinear transformations into convolutional operations, which improves nonlinear representation capability but also increases computational cost.

ConvKAN Efficient achieves the best classification accuracy among the 3 deep learning models. Compared with standard ConvKAN, it reduces the training time per epoch from 2.5017 to 2.1972 s and decreases the single-sample inference time from 0.003761 to 0.002600 s, corresponding to reductions of approximately 12.17% and 30.87%, respectively. These results suggest that the optimization strategies adopted in ConvKAN Efficient improve the computational process of KAN-based convolution, enabling more efficient training and inference while preserving strong nonlinear modeling capability.

ConvKAN Efficient provides a favorable balance between classification performance and computational cost. Although KAN-based convolution remains more computationally demanding than standard CNNs, the efficiency-optimized design of ConvKAN Efficient reduces the overhead of standard ConvKAN and achieves superior depression recognition performance.

### Confusion Matrix Evaluation

The confusion matrix provides a detailed comparison between the true labels and predicted labels, reflecting the classification accuracy, error distribution, and misclassification characteristics of each model in the depression recognition task (Figure 5). Compared with overall evaluation metrics such as accuracy and *F*_1_-score, confusion matrices offer more intuitive insights into how different models distinguish between those who have depression and healthy participants, thereby enabling a deeper analysis of model robustness and clinical applicability.

Among all evaluated methods, ConvKAN Efficient achieved the best classification performance, exhibiting the lowest numbers of both false positives and false negatives. Specifically, the model correctly identified 300 participants with depression as positive cases and 269 healthy participants as negative cases, while producing only 24 false positives and 7 false negatives. These results indicate that ConvKAN Efficient possesses strong discriminative capability for EEG-based depression recognition and can effectively reduce both missed diagnoses and false alarms.

From a clinical perspective, false negatives are particularly critical because patients with depression incorrectly classified as healthy individuals may fail to receive timely psychological intervention or treatment. The extremely low false negative count of ConvKAN Efficient demonstrates its strong sensitivity to depression-related EEG abnormalities, indicating high reliability for clinical screening applications. Meanwhile, the relatively low number of false positives suggests that the model also avoids excessive overdiagnosis, which is important for reducing unnecessary psychological burden and medical costs.

Compared with ConvKAN Efficient, the original ConvKAN model also achieved relatively strong classification capability, particularly in terms of false negative control, where only 12 participants with depression were misclassified. This finding suggests that the introduction of KAN-based nonlinear adaptive feature mapping significantly improves the model’s ability to capture depression-related EEG characteristics. However, ConvKAN produced 45 false positives, indicating that although the model is highly sensitive to depression-related patterns, it may occasionally overestimate abnormal EEG characteristics in healthy individuals. This phenomenon implies that the original ConvKAN architecture may possess relatively strong feature sensitivity but insufficient decision boundary refinement, leading to reduced specificity.

The CNN model achieved intermediate performance between traditional machine learning approaches and KAN-based architectures. Specifically, CNN produced 37 false positives and 29 false negatives, indicating limited capability in distinguishing complex EEG patterns associated with depression. Although CNN can effectively extract local spatial features through convolution operations, its fixed convolution kernels may struggle to adaptively model the highly nonlinear and nonstationary characteristics of EEG signals. Furthermore, EEG signals contain complex temporal dependencies and cross-channel interactions that conventional CNN architectures may fail to capture adequately. Consequently, CNN exhibited relatively balanced but less competitive classification performance.

Among the traditional machine learning models, LR and DT demonstrated relatively stable classification behavior. Their false positive counts were 27 and 22, respectively, indicating moderate discriminative ability for healthy participants. However, these models still produced relatively large numbers of false negatives compared with ConvKAN Efficient, suggesting insufficient sensitivity to depression-related EEG features. Since traditional machine learning approaches rely heavily on handcrafted statistical features and shallow decision mechanisms, they are inherently limited in modeling high-dimensional nonlinear EEG patterns.

LDA exhibited the weakest overall performance among all evaluated methods. The model generated 41 false negatives and 29 false positives, indicating substantial limitations in both sensitivity and specificity. This result may primarily stem from the strong linearity and Gaussian distribution assumptions of LDA, which are difficult to satisfy in practical EEG depression recognition scenarios. EEG signals are highly nonlinear, dynamic, and participant-dependent, making it challenging for LDA to construct sufficiently discriminative decision boundaries.

**Figure 5.**
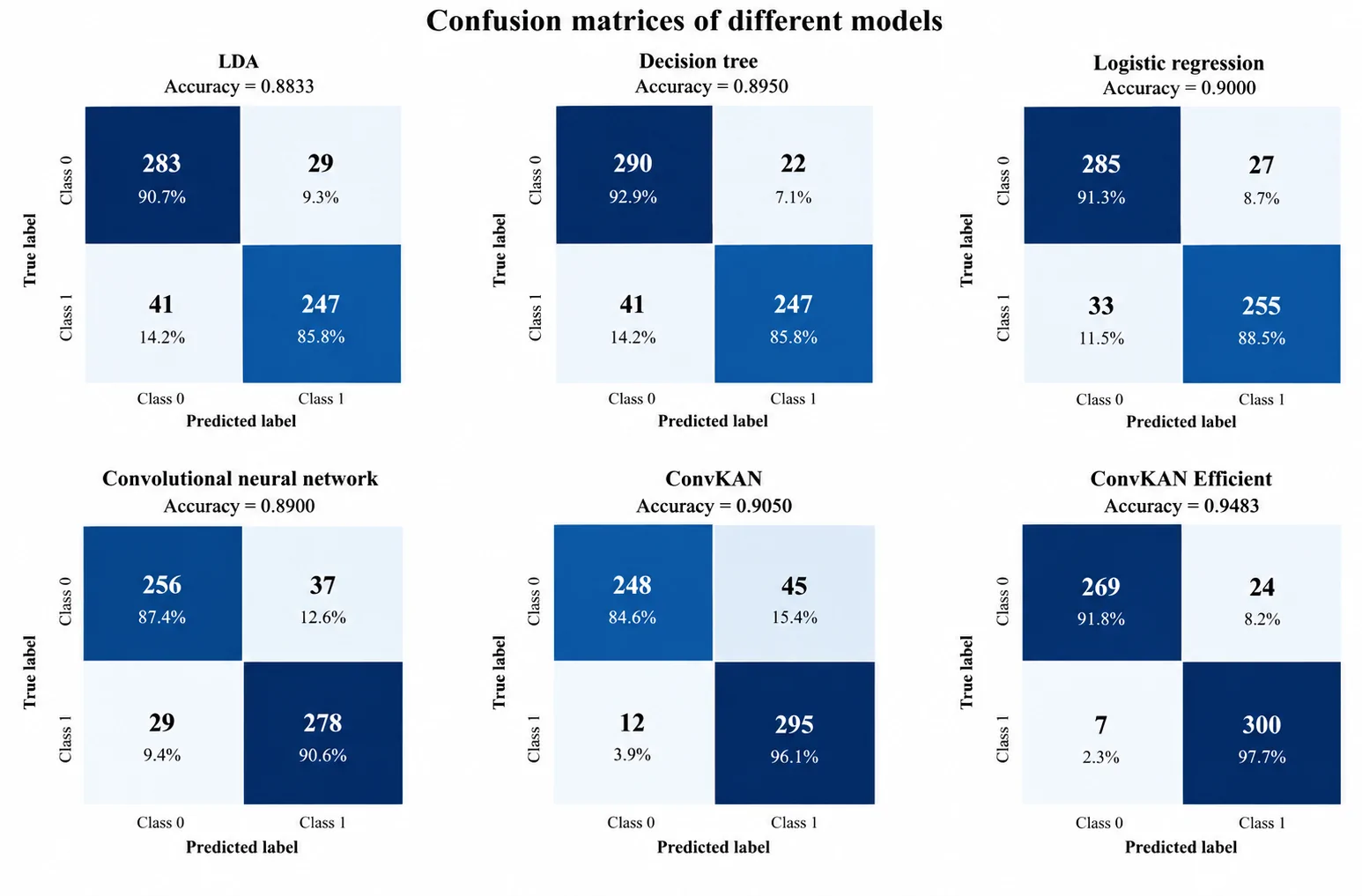
Confusion matrix of each model. CNN: convolutional neural network; DT: decision tree; LDA: linear discriminant analysis; LR: logistic regression.

Another important observation from the confusion matrix analysis is that KAN-based models consistently reduced false negative predictions compared with conventional CNN and traditional machine learning methods. This finding suggests that adaptive nonlinear feature transformation mechanisms are particularly effective for identifying subtle depression-related neural activity patterns embedded within EEG signals. Since depression-related EEG abnormalities may manifest as complex frequency-domain and temporal interactions rather than simple linear changes, KAN-based architectures provide stronger nonlinear approximation capability for modeling such neural dynamics.

The confusion matrix analysis further confirms the superiority of ConvKAN Efficient in EEG-based depression recognition. The model not only achieved the highest overall classification accuracy but also maintained balanced sensitivity and specificity, effectively minimizing both false positive and false negative rates. These characteristics are highly desirable for practical computer-aided depression screening systems, where reliable detection and stable generalization performance are essential. The results also demonstrate that incorporating KAN-based nonlinear adaptive feature learning into EEG classification frameworks can substantially enhance the discriminative capability of deep learning models for complex biomedical signal analysis.

### Receiver Operating Characteristic Curve Evaluation

The receiver operating characteristic (ROC) curve is a widely used evaluation tool for binary classification tasks and provides a comprehensive visualization of the trade-off between sensitivity and specificity under different decision thresholds. The AUC quantitatively reflects the overall discriminative capability of a classification model, where a larger AUC value indicates a stronger ability to distinguish between positive and negative samples. Compared with single-threshold metrics such as accuracy or *F*_1_-score, ROC analysis evaluates model performance across the entire threshold space, making it particularly suitable for assessing robustness and generalization capability in medical diagnosis applications. By plotting the ROC curves of each model and calculating the corresponding AUC values, a more comprehensive understanding of the strengths and limitations of different approaches for EEG-based depression recognition can be obtained (Figure 6).

**Figure 6.**
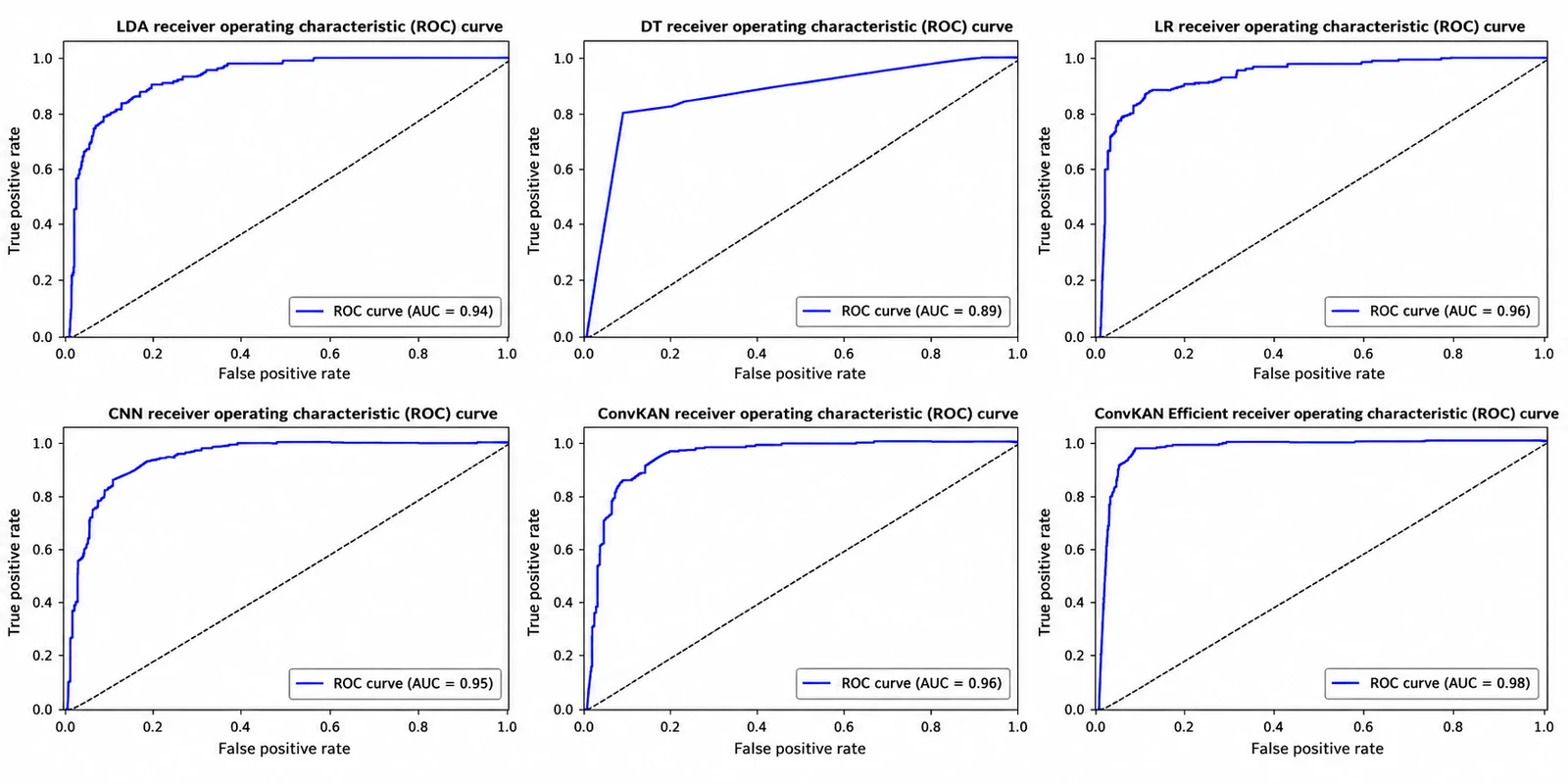
ROC curves of each model. AUC: area under the curve; CNN: convolutional neural network; DT: decision tree; LDA: linear discriminant analysis; LR: logistic regression; ROC: receiver operating characteristic.

Among the traditional machine learning approaches, LDA achieved the lowest AUC value of 0.89, indicating relatively limited discriminative capability for distinguishing participants with depression from healthy controls. This result is consistent with the previous confusion matrix analysis and further demonstrates that the linear assumptions underlying LDA are insufficient for modeling the highly nonlinear and dynamic characteristics of EEG signals. Since EEG features often exhibit complex interchannel correlations and nonlinear oscillatory behaviors, LDA may struggle to construct effective classification boundaries, resulting in reduced sensitivity and specificity across different thresholds.

The DT model achieved an AUC of 0.94, representing a considerable improvement over LDA. This finding suggests that hierarchical nonlinear feature partitioning can better capture complex EEG feature relationships compared with purely linear methods. By recursively splitting feature space into multiple decision regions, DT can partially model nonlinear interactions within EEG signals. However, despite its relatively strong classification capability, DT still showed fluctuations in classification stability, likely due to its sensitivity to local data distributions and tendency toward overfitting in limited-sample scenarios.

LR achieved an AUC of 0.96, outperforming both LDA and DT. This result demonstrates that, after appropriate EEG preprocessing and PSD feature extraction, the frequency-domain features possess relatively strong global statistical separability. The probabilistic decision mechanism of LR enables more stable boundary estimation compared with LDA, thereby improving overall classification performance. Moreover, the smooth and stable ROC curve of LR indicates relatively balanced sensitivity and specificity under varying decision thresholds, highlighting its robustness for EEG classification tasks.

Among the deep learning approaches, CNN achieved an AUC of 0.95, slightly lower than LR. Although CNNs possess strong automatic feature extraction capability, particularly in spatial feature learning, their performance advantage was not fully reflected in this EEG-based depression recognition task. This phenomenon may be attributed to several factors. First, EEG signals differ substantially from image data and contain complex temporal dependencies and nonlinear oscillatory patterns that conventional CNNs may not fully capture using fixed convolution kernels. Second, CNN architectures often require large-scale datasets to effectively learn representative deep features, whereas EEG depression datasets are typically limited in size. Consequently, CNN showed relatively good but not dominant classification capability.

The ConvKAN model achieved an AUC of 0.96, matching the performance of LR while outperforming CNN. This result highlights the effectiveness of integrating KAN-based adaptive nonlinear mapping mechanisms into convolutional architectures. Unlike traditional CNNs that rely on fixed linear convolution kernels, ConvKAN uses adaptive nonlinear transformations through KANLinear layers, enabling more flexible approximation of complex EEG feature distributions. The improved ROC performance indicates that ConvKAN can more effectively separate depression-related EEG patterns from normal neural activity across multiple classification thresholds.

Notably, ConvKAN Efficient achieved the highest AUC value of 0.98, significantly outperforming all other models. The ROC curve of ConvKAN Efficient remained consistently close to the upper-left corner of the coordinate system, indicating excellent sensitivity and specificity across different decision thresholds. This result demonstrates the strong discriminative capability and stable classification behavior of the evaluated ConvKAN Efficient model.

The superior ROC performance of ConvKAN Efficient can be attributed to several architectural improvements. First, the dynamic grid updating mechanism enables adaptive adjustment of spline representations according to EEG feature distributions during training, thereby improving nonlinear feature approximation capability. Second, optimized Kaiming weight initialization enhances training stability and facilitates more effective gradient propagation in deep architectures. Third, the incorporation of L1 regularization and entropy regularization helps suppress overfitting while improving feature sparsity and robustness. Collectively, these optimization strategies enhance both the representation capability and generalization performance of the model.

From a clinical application perspective, the high AUC value of ConvKAN Efficient is particularly important because it indicates strong reliability under different diagnostic thresholds. In practical depression screening systems, clinicians may adjust decision thresholds according to specific diagnostic requirements. A model with a consistently high ROC curve can maintain stable classification performance even when thresholds vary, thereby improving practical applicability and clinical flexibility.

Another important observation is that KAN-based models consistently achieved superior ROC performance compared with conventional CNN and traditional machine learning approaches. This finding further demonstrates that adaptive nonlinear modeling plays a critical role in EEG signal analysis. Depression-related neural activity patterns are often characterized by subtle nonlinear interactions across multiple frequency bands and brain regions. Therefore, models capable of adaptive nonlinear feature transformation are more effective for capturing these complex neural dynamics.

## Discussion

This study systematically investigated EEG-based depression classification using traditional machine learning methods, conventional CNN models, and KAN-enhanced deep learning architectures. Experimental results demonstrated that the investigated ConvKAN Efficient model consistently outperformed all baseline methods in terms of accuracy, precision, recall, *F*_1_-score, and AUC. These findings indicate that KAN-based nonlinear feature modeling can effectively enhance the representation capability of EEG signals and provide a promising framework for computer-aided depression assessment.

Compared with conventional machine learning methods such as LDA, DT, and LR, deep learning models exhibited stronger capability in learning discriminative EEG representations. Traditional machine learning approaches mainly rely on manually designed statistical features and linear decision boundaries, which may be insufficient for capturing the highly nonlinear and nonstationary characteristics of EEG signals. Although LR achieved relatively stable performance, its linear modeling capability limits its effectiveness when handling complex cross-channel EEG dependencies.

The conventional CNN model achieved only moderate performance in this study and did not significantly outperform several traditional machine learning models. This phenomenon may be attributed to the intrinsic characteristics of EEG signals. Unlike image data, EEG signals exhibit strong temporal variability, nonstationarity, and complex nonlinear dynamics. Standard CNN architectures mainly focus on local spatial feature extraction using fixed convolution kernels and therefore may struggle to capture adaptive nonlinear relationships across EEG channels and frequency-domain representations.

By incorporating KAN into convolutional operations, the proposed ConvKAN framework enhanced nonlinear feature learning capability and achieved improved classification performance compared with conventional CNNs. The spline-based adaptive mapping mechanism of KAN enables more flexible nonlinear transformations of EEG features, thereby improving the model’s ability to characterize subtle depression-related neural patterns. This suggests that KAN-based architectures may provide advantages in modeling the complex nonlinear distributions commonly observed in EEG signals.

ConvKAN Efficient further improved the performance of ConvKAN through dynamic grid updating, optimized weight initialization, and enhanced regularization strategies. The dynamic grid updating mechanism adaptively adjusts spline representations according to the distribution of input features, enabling more stable nonlinear approximation during training. Meanwhile, Kaiming initialization improves gradient propagation in deep architectures, while L1 and entropy regularization help suppress overfitting and enhance model robustness. These optimization strategies collectively contributed to the superior classification performance achieved by ConvKAN Efficient.

The experimental findings also demonstrate the potential clinical significance of the proposed framework. EEG-based depression assessment provides a noninvasive and objective approach that may complement conventional clinical interviews and self-report scales, which are often influenced by subjectivity and clinician experience. With further optimization and validation, KAN-based EEG analysis models may support early depression screening, computer-aided diagnosis, and mental health monitoring in clinical and community health care environments. In particular, optimized KAN-based nonlinear architectures such as ConvKAN Efficient may exhibit potential for deployment in wearable EEG systems and resource-constrained intelligent health care devices.

Despite the encouraging results, several limitations should be acknowledged. First, the EDRA dataset used in this study contains a relatively limited number of participants, which may restrict the generalization capability of the proposed model. Although participant-independent partitioning was adopted to prevent data leakage, larger multicenter EEG datasets are still necessary to further validate the robustness of the framework.

Second, this study focused primarily on frequency-domain PSD features. While PSD features effectively characterize spectral information in EEG signals, additional temporal, spatial, and functional connectivity features may further improve depression classification performance. Future studies may explore multimodal EEG feature fusion strategies to enhance representation capability.

Third, external validation across multiple public EEG depression datasets was not conducted in this work. Since EEG distributions may vary across acquisition devices, recording protocols, and participant populations, cross-dataset evaluation is essential for assessing real-world generalization performance.

Fourth, although ConvKAN Efficient achieved superior classification performance, the interpretability of KAN-based feature representations remains limited. Future research may integrate explainable AI techniques to identify the EEG regions and frequency components most strongly associated with depression-related neural activity.

Finally, although ConvKAN Efficient improves computational efficiency compared with the original ConvKAN framework, spline-based nonlinear operations may still introduce additional computational overhead relative to conventional CNNs. Further lightweight optimization and hardware-aware deployment strategies should therefore be investigated.

Future work will focus on several directions. First, multimodal fusion frameworks integrating EEG with speech, facial expression, or behavioral data may further improve depression assessment performance. Second, hybrid architectures combining KAN with transformer or graph neural network modules may provide stronger temporal and spatial modeling capability for EEG analysis. Third, lightweight and edge-deployable implementations should be explored to facilitate real-time mental health monitoring applications. In addition, explainable KAN-based frameworks may help improve clinical trustworthiness and promote the practical adoption of AI-assisted depression diagnosis systems.

Overall, the proposed ConvKAN Efficient framework demonstrates the feasibility and effectiveness of KAN-based nonlinear modeling for EEG depression classification. The results suggest that adaptive nonlinear feature learning may provide an important direction for future EEG-based AI research in mental health applications.

## Abbreviations

AUC: area under the curve
CNN: convolutional neural network
DT: decision tree
EEG: electroencephalogram
KAN: Kolmogorov-Arnold Networks
LDA: linear discriminant analysis
LR: logistic regression
LSTM: long short-term memory
MDD: major depressive disorder
PROMIS: Patient-Reported Outcomes Measurement Information System
PSD: power spectral density
ROC: receiver operating characteristic
SNN: spiking neural network
SVM: support vector machine
SVM-RFE: support vector machine–recursive feature elimination
TF-IDF: term frequency–inverse document frequency
UA-DAAN: uncertainty-aware dynamic adversarial adaptation network
XGBoost: Extreme Gradient Boosting
